# Mammographic microcalcifications and risk of breast cancer

**DOI:** 10.1038/s41416-021-01459-x

**Published:** 2021-06-14

**Authors:** Shadi Azam, Mikael Eriksson, Arvid Sjölander, Marike Gabrielson, Roxanna Hellgren, Kamila Czene, Per Hall

**Affiliations:** 1 Department of Medical Epidemiology and Biostatistics, Karolinska Institute, Stockholm, Sweden; 2 Department of Mammography, South General Hospital, Stockholm, Sweden; 3 Department of Oncology, South General Hospital, Stockholm, Sweden

**Keywords:** Cancer epidemiology, Breast cancer

## Abstract

**Background:**

Mammographic microcalcifications are considered early signs of breast cancer (BC). We examined the association between microcalcification clusters and the risk of overall and subtype-specific BC. Furthermore, we studied how mammographic density (MD) influences the association between microcalcification clusters and BC risk.

**Methods:**

We used a prospective cohort (*n* = 53,273) of Swedish women with comprehensive information on BC risk factors and mammograms. The total number of microcalcification clusters and MD were measured using a computer-aided detection system and the STRATUS method, respectively. Cox regressions and logistic regressions were used to analyse the data.

**Results:**

Overall, 676 women were diagnosed with BC. Women with ≥3 microcalcification clusters had a hazard ratio [HR] of 2.17 (95% confidence interval [CI] = 1.57–3.01) compared to women with no clusters. The estimated risk was more pronounced in premenopausal women (HR = 2.93; 95% CI = 1.67–5.16). For postmenopausal women, microcalcification clusters and MD had a similar influence on BC risk. No interaction was observed between microcalcification clusters and MD. Microcalcification clusters were significantly associated with in situ breast cancer (odds ratio: 2.03; 95% CI = 1.13–3.63).

**Conclusions:**

Microcalcification clusters are an independent risk factor for BC, with a higher estimated risk in premenopausal women. In postmenopausal women, microcalcification clusters have a similar association with BC as baseline MD.

## Introduction

Breast microcalcifications are deposits of calcium in the breast tissue and appear as small bright spots on mammograms [1]. Microcalcifications play a crucial role in breast cancer screening, particularly so for the non-palpable breast cancers [2], and are present in approximately one-third of all malignant lesions detected at screening mammography [3, 4]. They are more commonly found in ductal carcinoma in situ [3] than in invasive breast cancers [5]. Despite the well-recognised association between microcalcifications and breast cancer, previous studies have limitations, such as the inability to exclude microcalcifications without a malignant potential [6], using a crude, qualitative and reader-dependent measure of mammographic features (Breast Imaging-Reporting and Data System, BI-RADS, score) [7–10], inability to investigate the joint effect of mammographic density and microcalcifications [6–9], not including invasive breast cancer [7, 8], not taking menopausal status into consideration [6] and using case–control rather than prospective cohort designs [6, 9].

Only one risk prediction model for breast cancer has included microcalcifications and masses [11]. All other risk prediction models use lifestyle factors [12], family history of breast cancer [13], genetic determinants [14] or a combination of these factors together with mammographic density to predict the risk of developing breast cancer [15].

In this study, we were able to address some limitations in previous studies. We used the unique prospective Karolinska Mammography Project for Risk of Breast Cancer (KARMA) cohort [16] to investigate the association between microcalcification clusters, and risk of overall and subtype-specific breast cancer. We examined if baseline mammographic density influenced the association between microcalcification clusters and the risk of breast cancer. We presented the results stratified by menopausal status. Further, we studied the association between uneven distribution (asymmetry) of microcalcification clusters between the breasts and breast cancer risk and how mammographic density influenced the association between microcalcification clusters and the risk of breast cancer.

## Materials and methods

### Study population

**Fig. 1:**
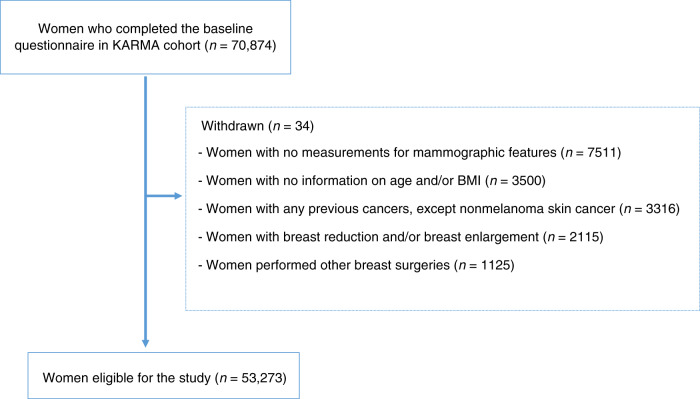
Flow chart describing the exclusion criteria for 70 874 women in KARMA cohort. Reasons for exclusions of participants in the Karolinska Mammography Project for Risk Prediction of Breast Cancer (KARMA) cohort. BMI body mass index.

KARMA is a population-based prospective screening cohort, which includes 70,874 women who were invited when conducting either screening (as part of the national mammography screening programme in Sweden) or clinical mammography at four hospitals in Sweden, from January 2011 to March 2013 [16]. Not all women were included in the analyses and the reasons for exclusions are given in Fig. 1. The final study included 53,273 women aged 30–80 years. To identify women with breast cancer, we linked the records of women within the KARMA cohort to the nationwide Swedish cancer registry. All KARMA cohort participants signed informed consent; at a later stage, we excluded *n* = 34 women who were asked to be removed from the study. The ethical review board of Karolinska Institutet approved the study.

### Measurement of mammographic features

Negative, non-diagnostic, mammograms were used when analysing microcalcification clusters and mammographic density. Digitally processed mammograms were collected and analysed (vendors General Electric, Philips, Sectram Hologic, Siemens) [11]. All women included in the KARMA cohort had a mammogram within 3 months from the study entry. To measure microcalcification clusters, raw mammograms from the mediolateral oblique (MLO) and craniocaudal (CC) views of the left and right breasts were collected. The CAD system used for the identification of microcalcification clusters (iCAD; M-Vu iCAD^®^, Nashua, USA) [17] is a Food and Drug Administration-approved class 3 device (PMA number P010038) with sensitivity of 92% [18]. The algorithm was designed to identify suspicious microcalcification clusters with a malignant morphology as defined by the BI-RADS 3–5 scores [18, 19] (Supplementary Methods). The microcalcification clusters were based on individual microcalcifications less than 1 mm of size. All individual microcalcifications within one millimetre from each other formed a mini cluster. All mini clusters within 5 mm of each other formed the main cluster. The main clusters were referred to as microcalcification clusters [20]. Hereafter, suspicious microcalcification clusters are just referred to as microcalcification clusters. The total (over both breasts) number of clusters and their asymmetry (difference in clusters between breasts) were treated as a continuous variable in the analysis, and categorised as (0, 1–2 and ≥3 clusters). We have used a similar method of detecting microcalcification clusters as in our previous publication [21] and illustrated how microcalcification clusters are marked on craniocaudal views using the iCAD software [21]. We used microcalcification clusters rather than single microcalcifications since clusters are more likely a sign of cancer [22, 23].

Using the STRATUS method, mammographic density was measured in each breast and the average dense area (cm^2^) and average percent density (%) over the left and right breasts were used [24]. STRATUS measures the mammographic dense area and the breast area and calculates the percent density from these measures. STRATUS was validated recently in two independent cohorts [25]. Also, the reproducibility of STRATUS was previously investigated [26, 27]. STRATUS is a fully automated tool developed to analyse digital and analogue images using an algorithm that measures density on all types of images, regardless of vendor. STRATUS measures the mammographic dense area and the breast area and calculates the percent density from these measures [24]. We chose to present the main results using the dense area since it is less influenced by body mass index (BMI) [28, 29]. Nevertheless, for compatibility with other studies, we also presented the results using percent density. Mammographic dense area and percent mammographic density were categorised into quartiles (<10.0, 10.0–22.9, 23.0–40.9 and ≥41.0 cm^2^) and (<6.0, ≥6.0–18.0, >18.0–35.9 and ≥36.0%), respectively.

### Covariates

Participants completed a detailed web-based questionnaire on lifestyle factors at baseline and the following factors were included in the analysis: smoking status (never, former and current), alcohol consumption (gram/day), age at menarche (years), age at first birth (years), parity (yes, no), breastfeeding duration (months), oral contraceptive use (never, ever), menopausal hormone therapy use (never, former and current), first-degree family history of breast cancer (no, yes) and menopausal status (pre- or postmenopausal). Women reporting no natural menstruation over the past 12 months before study entry or no menstruation due to oophorectomy were considered postmenopausal. Women with missing information on menstruation status or having no menstruation due to gynaecological surgeries other than oophorectomy were considered premenopausal if they were age 50 years or younger and postmenopausal if older than 50 years.

### Statistical analyses

Cox proportional hazard regression was used, with age as the underlying timescale, to estimate the association between microcalcification clusters and their asymmetry with the risk of breast cancer. These models were adjusted for BMI (continuous), baseline mammographic density (continuous), smoking status (categorical), alcohol consumption (continuous), age at menarche (continuous), age at first birth (continuous), number of children (continuous), breastfeeding duration (continuous), oral contraceptive use (categorical), menopausal hormone therapy use (categorical) and family history of breast cancer (categorical). In addition, we also investigated the association between baseline mammographic density and risk of breast cancer while adjusting for all the above-mentioned potential covariates. Hazard ratios (HRs) and 95% confidence intervals (CIs) were reported. The proportional hazard assumption was tested using the Schoenfeld residual test, and no major model violation was observed. We repeated the analyses, allowing for interaction between microcalcification clusters and mammographic density, to study how these jointly influence breast cancer risk. A global test was used to determine the presence of interaction. Logistic regression was used to investigate the association between the presence of microcalcification clusters and breast cancer tumour characteristics (in situ vs. invasive and oestrogen receptor (ER)-positive vs. ER-negative), for women who developed breast cancer during follow-up, while adjusting for potential confounders. For this analysis, microcalcification clusters were categorised as 0 and ≥1. All statistical tests were two-sided.

The Spearman correlation test was used to assess the correlation between the total number of microcalcification clusters and their asymmetry. All statistical analyses were performed with R version 3.6.1. *P* values, obtained from two-sided Wald/maximum likelihood ratio tests, of less than 0.05, were considered statistically significant.

## Results

### Baseline characteristics

A total of 676 women were diagnosed with breast cancer (Table 1). The mean (SD) follow-up time was 5.4 (0.6) years. For breast cancer cases, the median number of years between the last negative mammogram and the date of diagnosis was 2.8 years. Women with ≥3 clusters were older, had a greater mean mammographic dense area and percent density, more likely to use menopausal hormone therapy and to have a first-degree relative diagnosed with breast cancer (Table 1).

**Table 1:** Characteristics of 53,273 women included in the study separated by the number of microcalcification clusters.

Characteristics	Total no. (%)	Microcalcification clusters, no. (%)		
		0	1–2	≥ 3
No. of women (%)	53,273	44,088 (82.7)	7017 (13.1)	2150 (4.0)
Baseline age, mean (SD), y	54.1 (9.7)	53.3 (9.6)	57.4 (9.7)	59.2 (10.0)
BMI, mean (SD), kg/m^2^	25.1 (4.1)	25.2 (4.1)	24.9 (4.1)	24.3 (3.8)
*Menopausal status, (%)*				
Premenopausal	24,537 (46.0)	21,595 (48.9)	2325 (33.1)	609 (28.3)
Postmenopausal	28,736 (53.9)	22,493 (51.0)	4692 (66.8)	1541 (71.6)
Mammographic area density (cm^2^) at baseline, mean (SD)	28.3 (23.8)	28.1 (23.5)	29.2 (24.4)	33.3 (25.8)
*Mammographic area density (cm*^*2*^*), (%)*				
<10.0	13,188 (24.7)	11,414 (25.8)	1706 (24.3)	399 (18.5)
10.0–22.9	13,185 (24.7)	10,556 (24.0)	1688 (24.0)	489 (22.7)
23.0–40.9	13,185 (24.7)	11,090 (25.0)	1780 (25.3)	574 (26.7)
≥41.0	13,186 (24.7)	10,595 (24.0)	1774 (25.0)	658 (30.6)
Missing	532 (1.0)			
Mammographic percent density (%) at baseline, mean (SD)	23.0 (19.4)	22.6 (19.3)	23.5 (19.8)	27.2 (20.5)
*Mammographic density (%)*				
<6.0	13,184 (24.7)	10,783 (24.4)	1623 (23.1)	384 (17.8)
≥6.0–18.0	13,187 (24.7)	11,171 (25.3)	1782 (25.3)	474 (22.0)
>18.0–35.9	13,184 (24.7)	11,179 (25.3)	1766 (25.1)	574 (26.7)
≥36.0	13,186 (24.7)	10,522 (23.8)	1777 (25.3)	688 (32.0)
Missing	532 (1.0)			
*Smoking status, (%)*				
Never	25,386 (47.6)	20,964 (47.5)	3316 (47.2)	1097 (51.0)
Former	20,912 (39.2)	17,301 (39.2)	2819 (40.1)	786 (36.5)
Current	6236 (11.7)	5224 (11.8)	776 (11.0)	234 (10.8)
Missing	739 (1.38)			
Alcohol consumption (g/day), mean (SD)	7.1 (8.5)	8.7 (8.6)	9.0 (8.9)	9.0 (9.5)
Missing (%)	1240 (2.3)			
Age at menarche, mean (SD)	13.0 (1.4)	13.0 (1.4)	13.1 (1.4)	13.1 (1.4)
Missing (%)	1615 (3.0)			
Age at first birth, mean (SD)	27.7 (5.2)	27.4 (5.2)	26.4 (5.1)	26.1 (5.3)
*Parity*				
Yes	45,836 (86.0)	37,952 (86.0)	6023 (85.8)	1846 (85.8)
No	6644 (12.4)	5503 (12.4)	872 (12.4)	267 (12.4)
Missing	793 (1.4)			
Number of children, mean (SD)	1.9 (1.0)	2.18 (0.8)	2.23 (0.8)	2.24 (0.8)
Missing (%)	7462 (14.0)			
Breastfeeding duration (months), mean (SD)	18.8 (10.0)	19.3 (9.7)	19.1 (9.9)	19.1 (9.6)
Missing (%)	2809 (5.2)			
*Oral contraceptive use*				
Never	7512 (14.1)	5815 (13.1)	1247 (17.7)	447 (20.7)
Ever	44,441(83.4)	37,245 (84.4)	5546 (79.0)	1636 (76.0)
Missing	1320 (2.4)			
*MTH use (%)*				
Never user	39,960 (75.0)	33,526 (76.0)	4969 (70.8)	1451 (67.4)
Former user	7373 (13.8)	5780 (13.1)	1163 (16.5)	429 (20.0)
Current user	1879 (3.5)	1519 (3.4)	263 (3.7)	97 (4.5)
Missing	4061 (7.6)			
*Family history of breast cancer (%)*				
No	44,422 (83.3)	36,944 (83.7)	5737 (81.7)	1728 (80.3)
Yes	7211 (13.5)	5801 (13.1)	1045 (14.8)	361 (16.7)
Missing	1640 (3.0)			
*Breast cancer status (%)*				
No	52,597 (98.7)	43,628 (98.9)	6867 (97.8)	2084 (96.9)
Yes	676 (1.2)	460 (1.0)	150 (2.13)	66 (3.0)
Person-years	291,788	241,689	38,323	11,672

*BMI*   body mass index, *MHT*   menopausal hormone therapy, *SD*   standard deviation.

*P value for *t* test of means or chi-square test of proportions between women with and women without breast cancer, tests were performed at the two-sided 0.05 significance level.

### Mammographic features and risk of breast cancer

Overall, each additional microcalcification cluster was associated with 20% increased risk of breast cancer in all women (hazard ratio (HR = 1.20; 95% CI = 1.13–1.28)) (Table 2). Women with ≥3 microcalcification clusters had an overall 2-fold increased risk of breast cancer compared to women with no clusters (hazard ratio (HR = 2.17; 95% CI = 1.57–3.01)), after adjusting for potential confounders (Table 2). The estimated risk was more pronounced in premenopausal women (HR = 2.93; 95% CI = 1.67–5.16). Similar results were seen for the asymmetry of clusters and risk of breast cancer (Table 2).

**Table 2:** HRs of breast cancer risk in relation to microcalcification clusters, their asymmetry and mammographic dense area.

Mammographic features	All women (*N* = 53,273, breast cancer cases =676)			Premenopausal women (*N* = 24,537, breast cancer cases =239)			Postmenopausal women (*N* = 28,736, breast cancer cases = 437)		
	Person- years	No. of breast cancer	HR (95% CI)^*^	Person- years	No. of breast cancer	HR (95% CI)^*^	Person- years	No. of breast cancer	HR (95% CI)^*^
*No. of microcalcification clusters*^†^									
0	241,689	460	1.00 (Reference)	118,806	184	1.00 (Reference)	122,882	276	1.00 (Reference)
1–2	38,323	150	1.86 (1.49–2.33)	12,789	35	1.62 (1.05–2.51)	25,534	115	1.91 (1.50–2.51)
≥3	11,672	66	2.17 (1.57–3.01)	3321	20	2.93 (1.67–5.16)	8351	46	1.94 (1.28–2.84)
Microcalcification clusters (continuous)^†^			1.20 (1.13–1.28)			1.28 (1.15–1.43)			1.18 (1.09–1.27)
Asymmetry of microcalcification clusters^†^									
0	245,519	482	1.00 (Reference)	119,879	187	1.00 (Reference)	125,639	295	1.00 (Reference)
1–2	40,858	159	1.82 (1.47–2.25)	13,432	42	1.85 (1.24–2.75)	27,425	117	1.80 (1.39–2.30)
≥3	5308	35	2.16 (1.36–3.41)	1605	10	2.62 (1.15–6.00)	3702	25	1.97 (1.12–3.40)
Asymmetry of microcalcification clusters (continuous)^†^			1.29 (1.18–1.40)			1.37 (1.19–1.60)			1.24 (1.12–1.37)
Mammographic dense area (cm^2^)									
<10.0	73,842	133	1.00 (Reference)	17,861	12	1.00 (Reference)	55,980	121	1.00 (Reference)
10.0–22.9	69,755	156	1.62 (1.23–2.12)	25,225	35	2.17 (1.00–4.70)	44,530	121	1.63 (1.22–2.19)
23.0–40.9	73,838	176	1.87 (1.40–2.50)	40,445	57	2.20 (1.03–4.70)	33,393	119	2.03 (1.47–2.80)
≥41.0	71,427	207	2.75 (2.06–3.68)	50,286	133	4.50 (2.17–9.27)	21,140	74	2.00 (1.35–2.88)

*CI*   confidence interval, *HR*   hazard ratio.

*Adjusted model: body mass index (continuous), smoking status (categorical), alcohol consumption (continuous), age at menarche (continuous), age at first birth (continuous), number of children (continuous), breastfeeding duration (continuous), oral contraceptive use (categorical), menopausal hormone therapy use (categorical), and family history of breast cancer (categorical) at baseline.

^†^Adjusted model: additionally, for baseline mammographic density (continuous).

Women in the highest-density category (>41.0 cm^2^) had a nearly threefold higher risk of breast cancer (HR = 2.75; 95% CI = 2.06–3.68) compared to those with the lowest density (<10.0 cm^2^) after adjustment for potential confounders (Table 2). The results were more pronounced among premenopausal women (HR = 4.50; 95% CI = 2.17–9.27) (Table 2). Similar results were found using mammographic percent density (Supplementary Table 1).

Women with no microcalcification clusters and the lowest baseline mammographic dense area (<10.0 cm^2^) were used as the reference when testing for the interaction between microcalcifications and mammographic density (Table 3). There was a two-time higher risk of breast cancer when contrasting women with no microcalcification clusters to women with ≥3 clusters, regardless of their baseline mammographic density. No interaction effect between microcalcification clusters and baseline mammographic density on the risk of breast cancer was found (*P*_interaction_ = 0.65). Similar results were seen when using mammographic percent density (Supplementary Table 2).

**Table 3:** HRs of breast cancer risk in relation to number of microcalcification clusters and mammographic dense area.

Microcalcification clusters	No. of BC	MD area <10.0 HR (95% CI)*	No. of BC	MD area 10.0–22.9 HR (95% CI)*	No. of BC	MD area 23.0–40.9 HR (95% CI)*	No. of BC	MD area >41.0 HR (95% CI)*	*P* value of interaction^†^
									0.65
0	91	1.00 (Reference)	105	1.50 (1.08–2.09)	123	1.90 (1.35–2.66)	140	2.72 (1.93–382)	
1–2	33	2.26 (1.43–3.55)	39	3.52 (2.32–5.33)	34	3.00 (1.75–4.58)	45	4.17 (2.64–6.58)	
≥3	9	2.06 (1.00–4.13)	14	3.71 (1.95–7.05)	10	4.24 (2.28–7.90)	29	5.81 (3.28–10.27)	

*BC*   breast cancer, *MD*   mammographic density, *CI*   confidence interval, *HR*   hazard ratio.

*Adjusted model: body mass index (continuous), smoking status (categorical), alcohol consumption (continuous), age at menarche (continuous), age at first birth (continuous), number of children (continuous), breastfeeding duration (continuous), oral contraceptive use (categorical), menopausal hormone therapy use (categorical), and family history of breast cancer (categorical).

^†^*P* value from two-sided Wald test for interaction term.

The table includes 672 breast cancer patients since measures of mammographic density were missing in four patients.

The presence of microcalcification was significantly associated with in situ breast cancer (OR = 2.03; 95% CI = 1.13–3.36) (Table 4). Microcalcification clusters were not associated with ER status.

**Table 4:** Association between microcalcification clusters and tumour characteristics.

Tumour characteristics	Number of women (*N*, %)	All women OR (95% CI)*	*P* value^†^
*Invasiveness*			
Invasive	576 (85.2)	1.00 (Reference)	Ref.
In situ	90 (13.3)	2.03 (1.13–3.63)	0.01
*Oestrogen receptor status*^‡^			
Positive	305 (45.1)	1.00 (Reference)	Ref.
Negative	264 (39.0)	0.73 (0.46–1.15)	0.18

*CI*   confidence interval, *OR*   odds ratio, *Ref.* reference.

*Adjusted model: age (continuous), body mass index (continuous), baseline mammographic density (continuous), smoking status (categorical), alcohol consumption (continuous), age at menarche (continuous), age at first birth (continuous), number of children (continuous), breastfeeding duration (continuous), oral contraceptive use (categorical), menopausal hormone therapy use (categorical), and family history of breast cancer (categorical) at baseline.

^†^*P* value from two-sided Wald test.

^‡^Among women with invasive breast cancer only.

The correlation between the total number of microcalcification clusters and their asymmetry was ρ = 0.95 indicating a strong correlation between these two variables. The results in Tables 3 and 4 are therefore only including the total number of clusters.

## Discussion

Using a large prospective cohort, we found microcalcification clusters to be significantly associated with an increased risk of breast cancer. Based on our categorisation of microcalcification clusters and mammographic dense area, these two entities influenced the risk of breast cancer to the same extent in postmenopausal women. In premenopausal women, mammographic density had a more pronounced influence on breast cancer risk. We found no interaction between microcalcification clusters and mammographic density on the risk of breast cancer. Microcalcification clusters were significantly related to in situ breast cancer but were not associated with ER status.

Breast microcalcifications are common, the majority are benign, increase with age and are characterised by their morphology, size and distribution [30, 31]. The most crucial and difficult step in studying the association between microcalcification and breast cancer is the definition and measurement of microcalcifications. Microcalcifications are heterogeneous and range from benign alterations to markers of malignancy [32]. Some studies include microcalcifications with low malignant potential, such as arterial calcifications that are not associated with breast cancer, but are a potential surrogate marker of atherosclerotic cardiovascular disease [33]. Other studies have used the Breast Imaging-Reporting and Data System (BI-RADS) score [7–10], which is a qualitative and reader-dependent measure of mammographic features [34]. To reduce the subjectivity, we use a CAD system developed to mimic the BI-RADS classification and target microcalcifications classified as BI-RADS 3–5 [18].

Reassuringly, our finding that breast cancer risk increases with the number of microcalcification clusters has been shown before [6, 9]. The association between the number of microcalcification clusters and the risk of in situ breast cancer is also in agreement with previous studies [7, 8]. Some studies did not include invasive breast cancer [7, 8] which reduces the generalisability of the findings and hampers the use of the results for risk prediction modelling.

The biological mechanism behind calcium deposition in breast tissue is not clearly understood, but given the heterogeneity of microcalcifications, most likely several biological processes are involved [1, 35]. Epithelial–mesenchymal transition has been suggested as a plausible biological explanation for the formation of malignant microcalcifications [35, 36]. Epithelial–mesenchymal transition allows epithelial cells, normally attached to the basement membrane, to undergo several biochemical changes, including increased migratory capacity, invasiveness and production of the extracellular matrix [35]. It has been hypothesised that epithelial cells that acquire mesenchymal characteristics become capable of producing breast microcalcifications [35].

Mammographic density is a strong and established risk factor for breast cancer [37–39]. We did not see an interactive effect of microcalcification clusters and mammographic density. Counterintuitively, mammographic density decreases over age, despite being associated with an increased risk of breast cancer, a disease more common in older ages. In contrast, the number of microcalcification clusters increases over age and mimics the age distribution of breast cancer, that is, a slow increase during premenopausal age followed by a sharper upturn after menopause. The age-dependent prevalence of clusters is probably partly an effect of normal ageing since epithelial–mesenchymal transition increases with age [40]. It could be that microcalcification clusters will be recognised as a complementary risk factor for breast cancer. While mammographic density indicates a general risk of breast cancer [37–39], microcalcification has the potential to indicate not only where in the breast cancer will develop but also when it will emerge [6].

Our study had a number of limitations that should be emphasised. Information on breast cancer risk factors was based on a self-reported questionnaire and therefore is prone to information bias. However, a differential misclassification is unlikely since women were not aware of the presence of microcalcification clusters in their breasts at the time of assessment. We used an FDA-approved CAD software for identifying microcalcification clusters with malignant potential. Some of these microcalcifications were likely found in blood vessels and not in the breast tissue. However, given the quite substantial risk of breast cancer seen in women with microcalcification, we believe that the majority of identified calcifications were not breast arterial calcifications. The strengths of our study were the prospective population-based design, the number of women included, detailed information of the established breast cancer risk factors, including pre- and postmenopausal women, small proportion of missing data for the majority of the risk factors, having full access to mammograms for the measurement of mammographic density using the fully automated STRATUS tool and comprehensive reporting of incident breast cancer through nationwide registers.

## Conclusions

To conclude, we found that microcalcification clusters, with malignant potential, were an independent risk factor for breast cancer with a similar effect as mammographic density, at least in the postmenopausal part of life. Our study is the first comprehensive attempt using a clinically proved method to detect suspicious microcalcification clusters and to shed light on the risk of breast cancer associated with the presence of microcalcification clusters. Very little is known about the aetiology of microcalcifications. It is, therefore, safe to state that more research is needed to identify the predictors of mammographic microcalcifications and thereby possibly the mechanism behind the association between microcalcifications and breast cancer.

## Supplementary information

Supplementary Material

STROBE Statement

## Data Availability

Data can be made available upon reasonable request. The KARMA data access https://karmastudy.org/contact/data-access/.
