# Supplementary material for: Mammographic microcalcifications and risk of breast cancer
Source: Br J Cancer. 2021 Jun 14;125(5):759–65. doi: 10.1038/s41416-021-01459-x (PMC8405644; doi:10.1038/s41416-021-01459-x)
Supplement: Supplementary file 1 — Supplementary Material [file 41416_2021_1459_MOESM1_ESM.doc]

# Supplementary Methods

Microcalcifications were measured, in both the mediolateral oblique and craniocaudal views, using the iCAD Computer-Aided Detection (M-Vu CAD®, Nashua, USA), software. The software is an FDA approved, class 3 device (PMA number P010038), with reproducibility being a part of the approval criteria. The probability of repeating detection of calcifications from the same breasts scanned on different mammography machines was 95.5% (+/- 3.4%) per image and 99.0% (+/-2.3%) per case. Previous results showed that the iCAD software is achieving a sensitivity of 92% in detecting suspicious microcalcification clusters (1, 2) with average specificity of 87% (3). Clusters were defined according to a well-established neural network algorithm implemented by the iCAD software (2, 4). The microcalcification clusters were based on individual microcalcifications less than 1 mm of size. All individual microcalcifications within one millimetre from each other formed a mini cluster. All mini clusters within 5 mm of each other formed a main cluster. The main clusters were referred to as microcalcification clusters (5). The software marks regions of interest on standard mammographic views to bring them to the attention of the radiologist.

# Supplementary References

1. iCAD. Breast Health Solutions: Advanced Cancer Detection Built on Artificial Intelligence. 2019-09-27.

2. Food and Drug Administeration - Summary of Safety and Effectiveness Data Mammoreader -iCAD.

3. Cole EB, Zhang Z, Marques HS, Edward Hendrick R, Yaffe MJ, Pisano ED. Impact of computer-aided detection systems on radiologist accuracy with digital mammography. AJR American journal of roentgenology. 2014;203(4):909-16.

4. Jeffrey C, WehnesJames P, MonacoDavid S, HardingJames H, PikeAnbinh T, HoLawrence M H. Microcalcification detection classification in radiographic images. United States Patent and Trademark 2014. https://patents.google.com/patent/US8855388B2/en.

5. Wehnes JM, J. Harding, D. Anbinh, P. Hanafy, L. . Microcalcification detection classification in radiographic images. United States Patent and Trademark. 2014.

**Supplementary Table 1.** HRs of breast cancer risk in relation to microcalcification clusters, their asymmetry and mammographic percent density

| Mammographic features |  | **All women**  (N=53,273, breast cancer cases =676) | |  | **Premenopausal women**  (N=24,537, breast cancer cases =239) | | | |  | **Postmenopausal women**  (N=28,736, breast cancer cases = 437) | | |
| --- | --- | --- | --- | --- | --- | --- | --- | --- | --- | --- | --- | --- |
|  |  |  |  |  |  |  |  |  |  |  |  |  |
|  | Person years | No. of breast cancer | HR (95% CI) ^*^ | Person years | | No.  of breast cancer | | HR (95% CI) ^*^ | | Person years | No. of breast cancer | HR (95% CI) ^*^ |
| No. of microcalcification clusters † |  |  |  |  | | |  |  | |  |  |  |
| 0 | 241,689 | 460 | 1.00 (Reference) | 118,806 | | | 184 | 1.00 (Reference) | | 122,882 | 276 | 1.00 (Reference) |
| 1-2 | 38,323 | 150 | 1.82 (1.46 to 2.27) | 12,789 | | | 35 | 1.56 (1.01 to 2.41) | | 25,534 | 115 | 1.92 (1.50 to 2.50) |
| ≥ 3 | 11,672 | 66 | 2.08 (1.50 to 2.88) | 3,321 | | | 20 | 2.72 (1.54 to 4.81) | | 8,351 | 46 | 1.90 (1.25 to 2.80) |
| Asymmetry. of microcalcification clusters † |  |  |  |  | | |  |  | |  |  |  |
| 0 | 245,519 | 482 | 1.00 (Reference) | 119,879 | | | 187 | 1.00 (Reference) | | 125,639 | 295 | 1.00 (Reference) |
| 1-2 | 40,858 | 159 | 1.77 (1.43 to 2.20) | 13,432 | | | 42 | 1.77 (1.19 to 2.64) | | 27,425 | 117 | 1.77 (1.37 to 2.28) |
| ≥ 3 | 5,308 | 35 | 2.06 (1.3to 3.25) | 1,605 | | | 10 | 2.40 (1.04 to 5.47) | | 3,702 | 25 | 1.93 (1.12 to 3.34) |
| Mammographic percent density (%) |  |  |  |  | | |  |  | |  |  |  |
| <6.0 | 69,829 | 127 | 1.00 (Reference) | 17,133 | | | 9 | 1.00 (Reference) | | 52,696 | 118 | 1.00 (Reference) |
| ≥6.0 – 18.0 | 73,635 | 171 | 1.66 (1.27 to 2.18) | 24,722 | | | 30 | 2.70 (1.14 to 6.34) | | 48,912 | 141 | 1.65 (1.23 to 2.21) |
| >18.0 – 35.9 | 74,272 | 189 | 2.17 (1.61 to 2.92) | 38,244 | | | 68 | 3.77 (1.62 to 8.77) | | 36,027 | 121 | 2.13 (1.54 to 3.02) |
| ≥36.0 | 71,126 | 185 | 3.35 (2.40 to 4.68) | 53,718 | | | 130 | 7.56 (3.21 to 17.7) | | 17,407 | 55 | 2.00 (1.24 to3.14) |

Abbreviations: CI= confidence interval; HR= hazard ratio

* Adjusted model: body mass index (continuous), smoking status (categorical), alcohol consumption (continuous), age at menarche (continuous), age at first birth (continuous), number of children (continuous), breast feeding duration (continuous), oral contraceptive use (categorical), menopausal hormone therapy use (categorical), and family history of breast cancer (categorical) at baseline

† Adjusted model: additionally, for baseline percent mammographic density (continuous)

**Supplementary Table 2.** HRs of breast cancer in relation to number of microcalcification clusters and mammographic percent density

| microcalcification clusters | No. of BC | Percent MD <6.0  HR (95% CI) * | No. of BC | Percent MD ≥6.0 -18.0  HR (95% CI) * | No. of BC | Percent MD >18.0-35.9  HR (95% CI) * | No. of BC | Percent MD ≥36  HR (95% CI) * | P value of interaction † |
| --- | --- | --- | --- | --- | --- | --- | --- | --- | --- |
|  |  |  |  |  |  |  |  |  | 0.33 |
| 0 | 89 | 1.00 (Reference) | 112 | 1.47 (1.06 to 2.04) | 142 | 2.16 (1.53 to 3.04) | 116 | 3.02 (2.05 to 4.43) |  |
| 1-2 | 31 | 2.03 (1.27 to 3.24) | 46 | 3.66 (2.45 to 5.47) | 31 | 3.19 (2.00 to 5.12) | 40 | 4.44 (2.68 to 7.36) |  |
| ≥3 | 8 | 1.28 (0.50 to 3.52) | 15 | 3.54 (1.86 to 6.73) | 16 | 3.74 (1.90 to 7.37) | 29 | 7.27 (3.50 to 15.12) |  |

Abbreviations: BC= breast cancer; MD= mammographic density; CI= confidence interval; HR= hazard ratio

* Adjusted model: Body mass index (continuous), smoking status (categorical), alcohol consumption (continuous), age at menarche (continuous), age at first birth (continuous), number of children (continuous), breast feeding duration (continuous), oral contraceptive use (categorical), menopausal hormone therapy use (categorical), and family history of breast cancer (categorical)

† P value from two-sided Wald test

Table includes 672 breast cancer patients since measures of mammographic density was missing in 4 patients
